# Transcriptomic Analysis of Hepatic Cells in Multicellular Organotypic Liver Models

**DOI:** 10.1038/s41598-018-29455-x

**Published:** 2018-07-27

**Authors:** Allison N. Tegge, Richard R. Rodrigues, Adam L. Larkin, Lucas Vu, T. M. Murali, Padmavathy Rajagopalan

**Affiliations:** 1 Department of Computer Science, Virginia Tech, Blacksburg, USA; 2 Department of Statistics, Virginia Tech, Blacksburg, USA; 3 Genetics, Bioinformatics, and Computational Biology Ph.D. Program, Virginia Tech, Blacksburg, USA; 4 Department of Chemical Engineering, Virginia Tech, Blacksburg, USA; 5 ICTAS Center for Systems Biology of Engineered Tissues, Virginia Tech, Blacksburg, USA; 6 Virginia Tech-Wake Forest School of Biomedical Engineering and Sciences, Virginia Tech, Blacksburg, USA

## Abstract

Liver homeostasis requires the presence of both parenchymal and non-parenchymal cells (NPCs). However, systems biology studies of the liver have primarily focused on hepatocytes. Using an organotypic three-dimensional (3D) hepatic culture, we report the first transcriptomic study of liver sinusoidal endothelial cells (LSECs) and Kupffer cells (KCs) cultured with hepatocytes. Through computational pathway and interaction network analyses, we demonstrate that hepatocytes, LSECs and KCs have distinct expression profiles and functional characteristics. Our results show that LSECs in the presence of KCs exhibit decreased expression of focal adhesion kinase (FAK) signaling, a pathway linked to LSEC dedifferentiation. We report the novel result that peroxisome proliferator-activated receptor alpha (PPARα) is transcribed in LSECs. The expression of downstream processes corroborates active PPARα signaling in LSECs. We uncover transcriptional evidence in LSECs for a feedback mechanism between PPARα and farnesoid X-activated receptor (FXR) that maintains bile acid homeostasis; previously, this feedback was known occur only in HepG2 cells. We demonstrate that KCs in 3D liver models display expression patterns consistent with an anti-inflammatory phenotype when compared to monocultures. These results highlight the distinct roles of LSECs and KCs in maintaining liver function and emphasize the need for additional mechanistic studies of NPCs in addition to hepatocytes in liver-mimetic microenvironments.

## Introduction

The liver participates in vital functions related to metabolism, detoxification, and mediation of the complex defense mechanisms within the body^1^. Hepatocytes, the primary cell type of the liver^1^, are responsible for several hepatic functions, including lipid metabolism, glucose homeostasis, and biotransformation of xenobiotics^1^. Hepatocytes are the most widely-studied type of cell in the liver^1^. In recent years, other types of non-parenchymal hepatic cells have received more attention, such as liver sinusoidal endothelial cells (LSECs) and Kupffer cells (KCs), especially to study the roles they play in the onset of hepatic fibrosis^2^, non-alcoholic fatty liver disease^3^ and cholestasis^4^. The liver has a stratified architecture where the Space of Disse ensures the separation of the hepatocytes from the LSECs^1^. This *in vivo* architecture and intercellular communications have been recognized as important for proper functioning of hepatic cells^1^.

Deciphering cell-type-specific signaling is challenging to achieve *in vivo*. Engineered hepatic tissues have been developed to overcome this challenge^5–13^. Early *in vitro* models of the liver included the hepatocyte monolayer (HM)^5–7^ and the collagen sandwich (CS)^5–7^ cultures comprised of primary rat hepatic parenchymal cells. HMs lose morphological characteristics in less than one week^5–7^. In contrast, CSs maintain their polarized morphology and hepatic functions, e.g., albumin and urea production, for up to six weeks^5–7^. Hepatocytes co-cultured with NPCs have been shown to better maintain morphology and function than HM and CS cultures^8^. We have previously reported 3D organotypic liver models^9,10,12^ that consist of hepatocytes separated from hepatic NPCs by a detachable, biopolymeric membrane that mimics the Space of Disse^12^. This *in vitro* system recapitulated the stratified sinusoidal architecture found *in vivo*, thus enabling intercellular communications arising from cell-cell contact and paracrine signaling. Each cell type maintained phenotype up to 16 days. Additionally, the 3D liver models elicited proliferation of each cell type, while still maintaining cellular ratios similar to those found *in vivo*^12^. Traditional 2D *in vitro* cultures^9^ did not exhibit these properties.

Genomewide transcriptional analysis has been used to better understand the functions of hepatic cell types. However, gene expression studies on the liver have largely focused on understanding transcriptional programs in hepatocytes^14–17^. HMs, compared to CSs, were shown to rapidly lose liver-specific functions, such as cytochrome P450 (CYP) activity and cholesterol metabolism, within 48 hours^14,15,17^. In addition, cultures derived from hepatic cell lines exhibited decreased expression of CYPs compared to those derived from primary hepatocytes^15,16^. A study of primary rat hepatocytes co-cultured with one of three fibroblasts cell lines sought to elucidate cell-cell interactions at a transcriptional level^18^. However, fibroblasts are an inadequate substitute for hepatic NPCs. Moreover, co-cultures do not emulate the stratification found *in vivo* in the liver. Previous analyses of LSEC transcriptomes^19–25^ have limitations since they have focused on comparing LSECs to other endothelial cells or have investigated perturbations after partial hepatectomy and viral infection. Transcriptional studies on KCs^26–29^ have identified specific genes and cues involved in macrophage functional specialization or have monitored these cells during liver regeneration. None of these studies have considered how hepatoctyes and NPCs may influence each other transcriptionally in a healthy environment.

In this study, we conduct a genome-wide transcriptomic analysis of two organotypic 3D liver models^12^ with the goal of uncovering the functional activities of NPCs. To the best of our knowledge, this is the first genome-wide gene expression study of hepatic NPCs cultured in an *in vitro* 3D liver model that includes multiple cell types. Through extensive bioinformatic analyses, we elucidate changes in perturbed pathways that occur within the 3D liver models as a result of the presence or absence of KCs. Based on these analyses of our transcriptional data, we show that the inclusion of KCs in the 3D liver models encourages LSECs to maintain a differentiated phenotype. Taken together, the results suggest that signaling between hepatocytes and NPCs results in cellular and organ level functions that indicate a healthy hepatic environment. We stress that our results are based on analyses of transcriptional data and protein interaction networks. Further mechanistic experiments are required in order to confirm them.

## Results

We measured expression profiles of hepatocytes, LSECs, and KCs in five different culture systems: 3D models containing all three cell types, 3D models containing only hepatocytes and LSECs, collagen sandwiches, hepatocyte monolayers, and KC monolayers. We note that the 3D liver models maintained phenotype through day 16. However, to be consistent with our previous work^12^, all samples from the 3D liver models were taken at day 12. KCs in monocultures were assessed at day 3. Henceforth, we refer to 3D liver models that are comprised of hepatocytes (H) and LSECs (L) as 3DHL and those that also include KCs (K) as 3DHLK.

### Each cell type has a distinct gene expression profile

**Figure 1:**
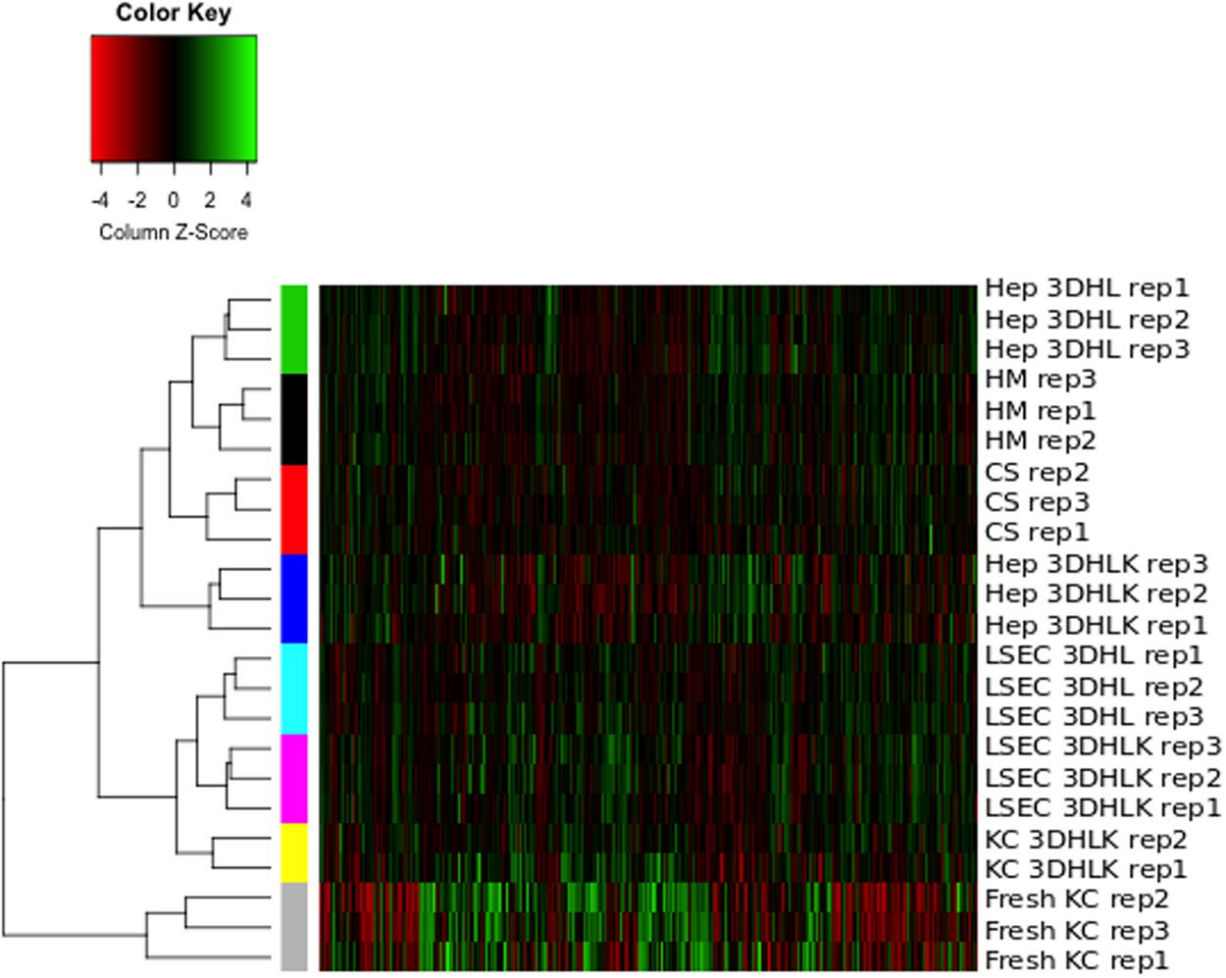
Hierarchical clustering of samples across cell types and culture conditions. Each column displays the expression values for one probe set. Each colored bar on to the left indicates the replicates of one cell type in one culture condition. From top to bottom, the samples are: hepatocytes in 3DHL models, HM, CS, and 3DHLK models; LSECs in 3DHL and 3DHLK models; KCs in 3DHKL models and monocultures of fresh KCs. We measured the expression profiles for the first seven groups on day 12 and for the last group on day 3.

We first sought to confirm the purity of our samples to ensure that no cross-contamination had occurred during the cell separation processes (See “Materials and Methods”). We performed hierarchical clustering of the transcriptomic samples using the probe-set-level expression data (Fig. 1). From this clustering, we observed the replicates for each cell-type and culture condition (e.g., LSECs from the 3DHLK model or the LSECs from the 3DHL model) clustered together. We also found the NPC samples clustered separately from the hepatocytes. Within the cluster containing the NPCs, the KCs from the 3DHLK model clustered distinctly from the LSECs in either 3D liver model. We evaluated the robustness of this clustering to different linkage and distance measures. The NPCs clustered separately from hepatocytes. Within the NPCs, the LSECs clustered separately from the KCs. KC monocultures clustered separately from all samples in the liver models, thus having the most distinct expression profiles. This separation was consistent across different linkage methods (single, complete, and average) and distance measures (Euclidean, Manhattan, and correlation)^30^.

We computed the average Euclidean distance among all hepatocyte samples, among all LSEC samples, between the KC samples in 3DHLK models, and across all pairs of cell types. The average distance from an LSEC sample to a KC sample was 81.59, which is at about 1.6 times the average distance among LSEC samples (55.02) or among KC samples (56.53). Hepatocytes were farther from both KCs and LSECs than KCs and LSECs were from each other.

These trends demonstrated that each cell type had a distinct and different expression pattern, and that the culture systems induced unique expression patterns in each cell type. Further, these data indicated that no contamination occurred during the cell separation process.

### LSECs in the 3DHLK model displayed expression patterns indicative of a differentiated phenotype

We used Gene Set Enrichment Analysis (GSEA)^31^ to identify gene sets exhibiting up- or down-regulation in the LSECs in the 3DHLK model compared to the LSECs in the 3DHL models (see “Materials and Methods”). GSEA identified 77 gene sets as enriched (*q*-value ≤ 0.01; Supplementary Table S1), of which 61 sets were up-regulated in LSECs in the 3DHLK model and 16 were down-regulated. Since GSEA considers each gene set individually, it may return statistically significant functions that annotate similar sets of genes. These redundant functions do not provide additional biological insights. In such cases, we discuss all related gene sets together. For each gene set, we examine genes in its leading edge (see “Materials and Methods”) to further study their relationships to hepatic phenotypes. We summarize our results in this and the next five sections.

Two phenotypic characteristics of differentiated LSECs are the presence of open (100–150 nm) fenestrae and their endocytotic activity^2^. GSEA identified several down-regulated gene sets related to these characteristics, specifically, the FAK Pathway (*q*-value 1.16 × 10^−3^, rank 37), Actin Cytoskeleton (*q*-value 7.10 × 10^−3^, rank 69), and Actin Filament Based Process (*q*-value 7.13 × 10^−3^, rank 70). These results suggest that FAK and actin signaling are decreased in the LSECs from the 3DHLK model when compared to the same cell type in the 3DHL model.

We focus on the FAK Pathway where GSEA identified 29 genes in its leading edge. The FAK pathway initiates with signals from FAK, Talin 1, and two integrins (Itga5 and Itgb1); the latter three participate in the leading edge of this gene set. This pathway includes three regulators of cell migration: Rho-kinase (Rock2)^32^, Ras homolog family member A (RhoA)^33^ and Ras-related C3 botulinum toxin substrate 1 (Rac1)^34^. All three proteins have been shown to control LSEC fenestration in rats. Specifically, the inhibition of Rock2 resulted in an increase in the number of fenestrae. Likewise, Rac1 has been associated with the contraction of fenestrae in rat LSECs^34^. FAK signaling has also been shown to regulate endocytosis^35^. Specifically, FAK-mediated activation of Endophilin A2 (Sh3gl1) inhibits endocytosis^35^. We observed decreased expression of Sh3gl1 in the LSECs from the 3DHLK model. Taken together, these transcriptional trends suggest that LSECs cultured in the 3DHLK liver model may have more and larger fenestrae and exhibit increased endocytotic activity compared to LSECs in the 3DHL model.

Nitric oxide (NO) is critical for the maintenance of LSEC function and differentiation^36^. When we compared LSECs to hepatocytes in the 3DHLK model (Supplementary Table S1), we observed up-regulation of ion channel transport, acetylcholine binding, and G protein coupled receptor (GPCR) ligand binding genes. To preclude the possibility that this up-regulation may be caused by a down-regulation of these processes in hepatocytes in 3DHLK models, we applied GSEA to compare these hepatocytes to the same cell type in 3DHL model (Supplementary Table S1). We observed that none of these gene sets were significant at 0.01 level in this comparison. Nicotinic cholinergic receptor alpha 3 and alpha 6 receptors and Muscarinic cholinergic receptor 1 participated in the leading edge of functions related to ion channel activity, neurotransmitter receptor activity, and GPCR activity. Upon activation by acetylcholine, these cholinergic receptors open ion channels, enabling changes in intracellular calcium ion levels^37,38^. Increased calcium levels have been shown to activate NO synthase in endothelial cells in a Calmodulin-dependent manner^39^. We observed that Calmodulin-dependent protein kinase IV (Camk4) was up-regulated in LSECs compared to hepatocytes, suggesting an increase in NO synthesis in LSECs. Two other well known markers of LSEC differentiation, Protein Tyrosine Phosphatase, Receptor Type C (CD45)^40^ and Von Willebrand Factor (VWF)^41^, were up-regulated in LSECs in 3DHLK models compared to 3DHL models. Taken together, these transcriptional results suggest LSECs in the 3DHLK model maintain more phenotypic characteristics of a differentiated cell type.

### A functional linkage network summarizes differences between LSECs in 3DHLK and in 3DHL systems

**Figure 2:**
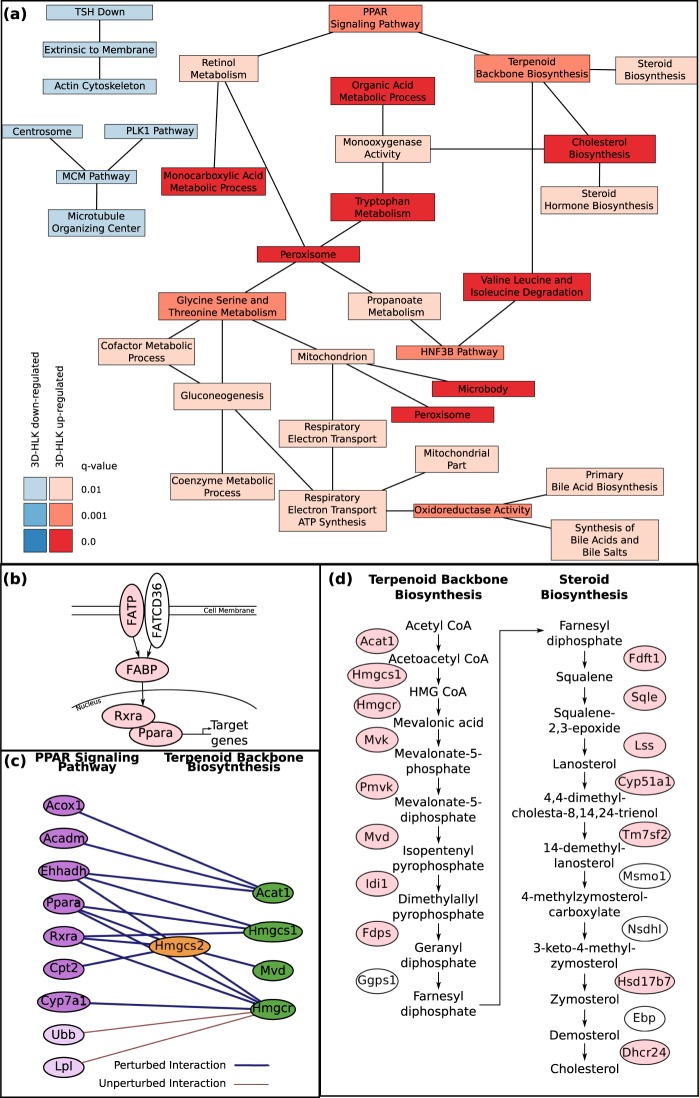
Results from enrichment and network analysis comparing expression profiles of LSECs in 3DHLK models to 3DHL models. (**a**) Network computed by the MCMC-BPN algorithm. Each node is an enriched gene set reported by GSEA. Each link connects two gene sets. Red nodes are up-regulated and blue nodes are down-regulated in LSEC from the 3DHLK model. (**b**) Protein-protein and regulatory interactions up-stream of Ppara in the PPAR Signaling Pathway. Proteins highlighted in pink were up-regulated in LSECs in the 3DHLK model. (**c**) Protein interactions underlying the link between the PPAR Signaling Pathway (purple nodes) and the Terpenoid Backbone Biosynthesis Pathway (green nodes) in the BPN displayed in (**a**). Nodes with a saturated color are in the leading edge. Note that Hmgcs2 is annotated to both pathways. (**d**) Reactions involved in cholesterol biosynthesis. Pink proteins belong to the leading edge of either the Terpenoid Backbone Biosynthesis or the Steroid Biosynthesis pathway.

Several of the enriched gene sets identified by GSEA shared leading edge genes. In addition, previous methods have demonstrated that genes annotated to one function may interact with those genes from another function in an orchestrated manner^42–44^. We applied the Markov chain Monte Carlo Biological Process Network (MCMC-BPN) algorithm^45^ to identify a non-redundant set of gene sets that connect to each other via interactions between perturbed genes (See “Materials and Methods”). We applied MCMC-BPN to 77 gene sets with a q-value at most 0.01, as determined by GSEA. Figure 2a displays the biological process network (BPN) formed by all links with a probability greater than or equal to 0.45. This threshold corresponds to the top 2.5% of possible functional links. This BPN contains 36 links among 34 of the top 77 gene sets. The BPN contains three connected components, two of which consist of only gene sets down-regulated in LSECs in the 3DHLK model, with the third including only up-regulated gene sets.

In the following sections, we discuss several of the top enriched functions identified by GSEA and the links between them in the BPN. Many of these up-regulated gene sets were related to the metabolism of fatty acids, bile acids and cholesterol. We first present leading edge genes from the Peroxisome Proliferator-activated Receptor (PPAR) signaling pathway. Next, we explore the link between PPAR signaling and cholesterol metabolism revealed by the BPN. Finally, we discuss how bile acid biosynthesis and fatty acid metabolism relate to these two gene sets.

### LSECs in the 3DHLK model exhibited increased PPAR signaling

LSECs in the 3DHLK model show up-regulation of genes in the PPAR Signaling Pathway (rank 1, *q*-value < 10^−5^) compared to those in the 3DHL model (Supplementary Table S1). GSEA assigned 26 of the 63 genes from this gene set to its leading edge, including Ppara (SNR 0.21, rank 206), a primary transcription factor, and its co-activators Rxra and Rxrg (SNR 0.18, rank 346; SNR 0.15, rank 586, resp.)^46–48^. PPARs are a group of three nuclear hormone receptors: PPARα, PPARδ and PPARγ. (We use ‘Ppara’ to name the gene and ‘PPARα’ for the protein product.) Only Ppara was up-regulated in LSECs in the 3DHLK model; Ppard and Pparg showed no significant difference in expression levels in LSECs between the two 3D cultures. The expression of Ppara in LSECs was elevated in comparison to its level in hepatocytes in the 3DHLK model (SNR: 0.14, rank 3866). Note that this gene was somewhat down-regulated in hepatocytes in the 3DHLK model compared to the 3DHL model (SNR: −0.11, rank 11745).

Up-stream of PPARα in the signaling pathway lie members of both the Fatty Acid Transport Protein (FATP)^49^ and Fatty Acid Binding Protein (FABP) families of proteins^50^. Consistent with the liver specific expression patterns reported in the literature^51–53^, we observed up-regulation of FATPs (Slc27a2 and Slc27a5, SNR: 0.14, rank 684; SNR: 0.26, rank 101, resp.) and FABPs (Fabp1/4/6, SNR: 0.49, rank 11; SNR: 0.12, rank 909; SNR: 0.10, rank: 1323, resp.), all of which participated in the leading edge (Fig. 2b). FATPs are transmembrane proteins involved in the uptake of long and very-long chain fatty acids into the cell^51,52^. FABPs act as an intracellular buffer by ushering ligands from FATPs to PPARα^54^. A murine study showed a physical interaction between the FABP1 and PPARα proteins *in vivo* in the liver and in cultured primary hepatocytes^50^. Note that the predominantly enterocyte specific FATP Slc27a4^55^ was highly down-regulated in LSECs.

Upon activation by ligands such as fatty acids and their derivatives, eicosinoids, and synthetic compounds, PPARα translocates from the cytoplasm to the nucleus^46^. In the nucleus, PPARα dimerizes with the co-activator retinoid X receptors (RXRα and RXRγ) and transcribes genes involved in lipid metabolism, gluconeogenesis and cell survival^56^. Both RXRα and RXRγ were up-regulated in LSECs in the 3DHLK model. These LSECs also showed increased expression of down-stream targets of PPARα. These targets are involved in cholesterol metabolism^57^ and bile acid biosynthesis (e.g., Cyp7a1 and Cyp8b1)^58–60^, fatty acid oxidation^61,62^ and amino acid degradation (e.g., Acadm and Ehhadh)^63^. We discuss the relevance of cholesterol metabolism, bile acid biosynthesis and fatty acid oxidation in the following sections.

### LSECs in the 3DHLK model regulate cholesterol metabolism

The MCMC-BPN analysis identified a link between PPAR Signaling Pathway and Terpenoid Backbone Biosynthesis gene set (link probability 0.90; Fig. 2a). Figure 2c displays the set of interactions representing this link; each interaction connects one gene in the PPAR Signaling Pathway to one gene in the Terpenoid Backbone Biosynthesis gene set. Sixteen out of the 18 interactions connected genes in the leading edges of these two gene sets. Six of these 16 interactions reflected the transcriptional regulation of 3-Hydroxy-3-Methylglutaryl-CoA Reductase (Hmgcr), 3-Hydroxy-3-Methylglutaryl-CoA Synthase 1 (Hmgcs1), and 3-Hydroxy-3-Methylglutaryl-CoA Synthase 2 (Hmgcs2) by PPARα and by RXRα^64^. The MCMC-BPN analysis also identified a link between Terpenoid Backbone Biosynthesis and both Cholesterol Biosynthesis and Steroid Biosynthesis (link probabilities 0.56 and 0.47, respectively). The Terpenoid Backbone Biosynthesis and Cholesterol Biosynthesis gene sets share seven genes that correspond to the set of reactions responsible for converting Acetyl-CoA to Farnesyl diphosphate^65^. Additionally, the Cholesterol Biosynthesis and Steroid Biosynthesis gene sets encompass genes responsible for the conversion of Farnesyl diphosphate to cholesterol^66^. Together, these three gene sets, all of which are up-regulated in LSECs in the 3DHLK model, annotate the set of reactions in cholesterol biosynthesis that convert Acetyl-CoA to cholesterol (Fig. 2d)^67–69^. These links in the BPN indicate that the PPAR Signaling Pathway may be activated and in turn induce the expression of Cholesterol biosynthesis genes in LSECs in the 3DHLK model.

**Figure 3:**
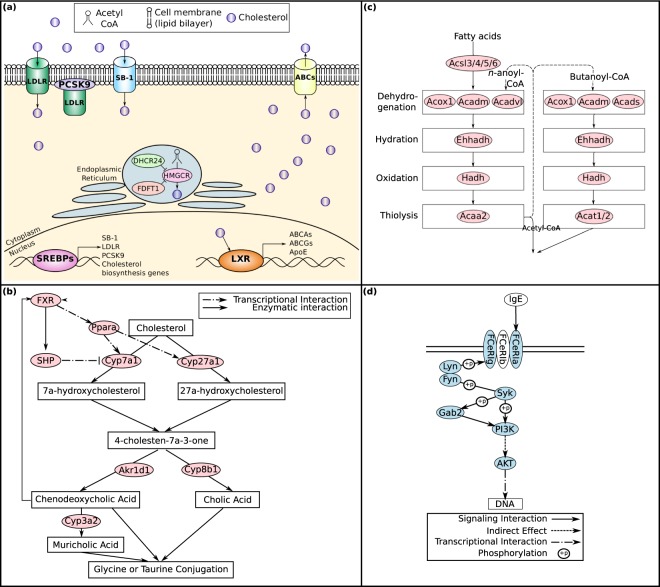
LSEC and KC signaling pathways in 3DHLK systems. (**a**) Pathway diagram of transcytosis of cholesterol in LSECs. (**b**) FXR-dependent feedback mechanisms involved in regulation of bile acid biosynthesis. Interactions represent both the positive feedback mechanism through Ppara and the negative feedback mechanism through SHP. Proteins highlighted in pink are up-regulated in LSECs in the 3DHLK model. (**c**) Fatty Acid Degradation and beta-oxidation pathway. Genes highlighted in pink were up-regulated in the LSECs in the 3DHLK model. (**d**) Diagram of the FC-ε RI Signaling Pathway. Proteins shown in blue are down-regulated in Kupffer cells from the 3DHLK model.

Further support for this observation comes from the significant up-regulation of the Reactome Cholesterol Biosynthesis gene set (rank 1, *q*-value < 10^−5^) in LSECs in the 3DHLK model versus LSECs in the 3DHL model (Supplementary Table S1 and Fig. 2a). However, cholesterol biosynthesis in the liver has mainly been attributed to hepatocytes^1^ and the involvement of LSECs has not been well documented. To further understand our results, we compared the expression of these genes in LSECs to hepatocytes in the 3DHLK model. We noted that the Cholesterol Biosynthesis gene set is not significantly perturbed when we compare Hepatocytes in the 3DHLK model to the same cell type in the 3DHL model (*q-*value 1). Cholesterol homeostasis occurs due to a combination of synthesis, transcytosis and metabolism^69–72^. Certain signaling events related to cholesterol homeostasis are triggered by cytoplasmic cholesterol concentrations (Fig. 3a). When cytoplasmic cholesterol levels are low, sterol regulatory element binding proteins (SREBPs) transcribe genes involved in *de novo* cholesterol biosynthesis^72^. In addition, activated SREBPs transcribe scavenger receptor B1 (Scarb1; SB-1), low-density lipoprotein receptor (LDLR), and proprotein convertase subtilisin/kexin type 9 (PCSK9), genes involved in the uptake of cholesterol^72^. Both LDLR and SB-1, which are responsible for the uptake of exogenous cholesterol^72^, show increased expression in LSECs (SB-1, SNR: 0.18, rank: 3121; LDLR, SNR: 0.49, rank: 7) suggesting that LSECs may participate in transcytosis of cholesterol. SB-1, the high density lipoprotein receptor, is known to be expressed abundantly in LSECs^70^. Interestingly, the SREBP transcriptional target PCSK9 plays a role in the clathrin-mediated endocytosis of LDLR^72^. In contrast, when cholesterol levels are high within a cell, the transcription factor liver X receptors (LXRs) are activated^72^. LXRs transcribe genes involved in the efflux of cholesterol, including ApoE, and the ATP-binding cassette transporters ABCA and ABCG family of transporters^72^. Though we observe down-regulation of ABCA1 and ABCG1 (SNR: −0.09, rank: 9463; SNR: −0.17, rank: 10834, resp.) in LSECs, the liver-specific ABCG5 displays increased expression (SNR: 0.192, rank: 2795). Another method for maintaining cholesterol homeostasis involves HMGCR, the rate-limiting step in cholesterol biosynthesis^73^. HMGCR may be post-translationally degraded by 24,25-dihydrolanosterol and squalene^73^. 24-Dehydrocholesterol Reductase (Dhcr24) and Farnesyl-Diphosphate Farnesyltransferase 1 (Fdft1), the two enzymes responsible for the synthesis of 24,25-dihydrolanosterol and squalene, respectively, exhibit increased expression in LSECs compared to hepatocytes (SNR: 2036, rank: 0.23; SNR: 0.39, rank: 232, resp.). Altogether, these trends in the transcriptional data suggest that LSECs in the 3DHLK models participate in cholesterol transcytosis as well as in the biosynthesis of this compound.

### LSECs in the 3DHLK model up-regulated genes involved in bile acid biosynthesis

The LSECs from the 3DHLK model, compared to those in the 3DHL cultures, showed up-regulation of genes associated with the Primary Bile Acid (BA) Biosynthesis gene set (*q*-value 4.93 × 10^−3^, rank 55, Supplementary Table S1 and Fig. 2a). As in the case of cholesterol biosynthesis, we compared the expression levels of these genes in LSECs to hepatocytes from the 3DHLK model, since only hepatocytes have been shown to produce BAs^58,74,75^. Using GSEA, we found no enrichment of gene sets related to BA biosynthesis (*q*-value > 0.01), suggesting similar expression levels among these genes in both LSECs and hepatocytes and supporting the possibility of bile acid biosynthesis in LSECs.

Synthesis of BAs is the primary means of cholesterol catabolism^58,76^ and can occur via the classical (neutral) or alternate pathways (Fig. 3b)^58^. In the initial step of BA formation in the classical pathway, Cytochrome P450, Family 7, Subfamily A, Polypeptide 1 (Cyp7a1) hydroxylates the C-7 carbon of cholesterol, and is the rate-limiting enzyme for determining the BA pool^58^. Cytochrome P450, Family 8, Subfamily B, Polypeptide 1 (Cyp8b1) leads to the generation of cholic acid (CA)^58^. Following the alternate pathway, Cytochrome P450, Family 27, Subfamily A, Polypeptide 1 (Cyp27a1) and Aldo-keto Reductase Family 1, Member D1 (Akr1d1) control the population of chenodeoxycholic acid (CDCA)^76^. We observed the up-regulation of Cyp7a1, Cyp8b1, Cyp27a1, and Akr1d1 in the leading edge of the Primary Bile Acid Biosynthesis gene set from the KEGG database when we compared LSECs from the 3DHLK model to those from 3DHL cultures. These results suggest that higher levels of CA and CDCA may be synthesized in LSECs in the presence of hepatocytes and KCs. CDCA has been found to induce FXR activity in humans and mice^76,77^, resulting in increased expression of PPARA^78^. These transcription-based results suggest that a feedback mechanism among PPARα, FXR, and BA biosynthesis occurs in LSECs when they are cultured with hepatocytes and KCs.

### LSECs in the 3DHLK model exhibited increased fatty acid metabolism

Six of the top 77 gene sets identified by GSEA were involved in fatty acid metabolism (Supplementary Table S1). These gene sets include Carboxylic Acid Metabolic Process, Organic Acid Metabolic Process and Monocarboxylic Acid Metabolic Process (rank 1, *q*-value < 10^−5^), Fatty Acid Oxidation (rank 14, *q*-value 3.34 × 10^−5^), Fatty Acid Metabolism (rank 17, *q*-value 1.36 × 10^−4^), and Fatty Acid Metabolic Process (rank 19, *q*-value 1.47 × 10^−4^). Our BPN included two gene sets related to fatty acids: Organic Acid Metabolic Process and Monocarboxylic Acid Metabolic Process. All six fatty acid related gene sets shared a core set of four genes in their leading edges: Cpt1a, Acadvl, Acadm, and Echs1, each being up-regulated in LSECs in the 3DHLK model compared to the 3DHL model (SNR ≥ 0.13; rank ≤ 833). Up-regulation of these gene sets suggests that LSECs in the presence of KCs may have increased fatty acid metabolism. In fact, LSECs are known to derive a large proportion of their energy (~45% of ATP) from fatty acid oxidation^79^.

Biosynthesis of cholesterol requires the availability of Acetyl-CoA^80^. Fatty acid oxidation and degradation provide one source of Acetyl-CoA^81^. These processes occur in either the peroxisome^82^ or the mitochondria^83^. For mitochondrial fatty acid oxidation, carnitine palmitoyltransferases (Cpt1a/Cpt2) first import fatty acids from the cytoplasm where their degradation occurs through β-oxidation (Fig. 3c)^84–87^. Acyl-CoA Dehydrogenase, Very Long Chain (Acadvl)^88^, Acyl-CoA Dehydrogenase, C-4 To C-12 Straight Chain (Acadm)^88^, Enoyl CoA Hydratase, Short Chain, 1 (Echs1)^89^, and Cpt1a^90^ participate in this process. Cpt1a regulates the rate-limiting step in rat hepatocytes^91^. All these genes highlighted in pink were up-regulated in the LSECs in the 3DHLK model. It is notable that Ppara, which is up-regulated in these LSECs, transcriptionally regulates Cpt1a and Acadm.

### KCs display an anti-inflammatory phenotype in the 3DHLK model

KCs, like many macrophages, exhibit plasticity in their phenotype ranging from pro- to anti-inflammatory characteristics^92,93^. To better understand their phenotype in the 3DHLK liver model, we compared their gene expression profiles in the 3DHLK model on day 12 to KC monocultures on day 3. GSEA identified 21 significant functions in the 3DHLK model (*q*-value < 10^−5^, Supplementary Table S1). GSEA identified four functions as up-regulated (*q*-value < 10^−5^) in KCs from the 3DHLK liver model. The remaining 17 gene sets were down-regulated. Of those functions showing increased expression in the KCs from the 3DHLK cultures, three were related to drug metabolism and phase II conjugation, and one involved genes found up-regulated in hepatocytes in response to LSECs^94^. The down-regulated gene sets related to inflammation and immune response.

We focus on gene sets that we can map to pathways, specifically the Fcε receptor signaling pathway in the KEGG database (Fig. 3d). The Fcε receptor is involved in allergic and immune responses, and is responsible for the release of histamines, leukotrienes, and cytokines after activation^95^. The Fcε receptor, specifically the Fcεr1γ subunit, is one mechanism responsible for the M2-to-M1 transition of macrophages^96^. The gene set for this signaling pathway contains 70 genes, 21 of which participate in the leading edge. In order to initiate Fcε signaling, immunoglobulin E (IgE) binds to Fcε receptors, which activate Lyn or Fyn, both Src Family Tyrosine Kinases^97,98^. Once activated, Lyn or Fyn phosphorylates the Fcεr1γ-chain subunit of Fcε receptor, resulting in the recruitment and subsequent activation of Syk by Lyn or Fyn^97,98^. We observed the down-regulation of Fcer1g, Fyn, Lyn, Syk, Gab2 and PI3K (PIK3R1/PIK3CD) in KCs in the 3DHLK models compared to monocultures. All seven of these genes were members of the leading edge of the Fcε-receptor signaling pathway. Additional characteristics of M1 macrophages include expression of pro-inflammatory cytokines such as tumor necrosis factor (TNF), IL6, IL12, and nitric oxide synthase 2, inducible (iNOS)^92^. TNF, which is transcriptionally up-regulated by Fcε receptor signaling, IL6, and iNOS exhibited decreased expression in KCs in the 3D model compared to those in the monoculture. In contrast, macrophages with an anti-inflammatory phenotype express IL10 protein, IL1 receptor agonist, and IL1 decoy receptor^99^, which were up-regulated in the 3D cultures. These trends in the transcriptional data support the conclusion that KCs in 3DHLK cultures show a more M2-like phenotype in comparison to freshly isolated KCs.

## Discussion

Hepatocytes are the most widely studied cell type in the liver. More recently, scientists have begun to understand the critical roles played by NPCs, especially LSECs and KCs, in modulating the function of healthy livers. In this paper, we have sought to discover transcriptional perturbations as a result of heterotypic communications among hepatic cell types in an organotypic liver model that maintained the phenotypes of each cell type for up to 16 days^12^. This model permits the separation of cell types thus enabling us to measure the transcriptome in each cell type individually. Thereby, we created the first-ever transcriptional dataset in hepatocytes and in two types of NPCs (LSECs and KCs) in an *in vitro* liver model. We noted that each cell type had a distinct expression profile and that each culture system induced unique expression patterns in each cell type. These results indicate that no contamination occurred during the cell separation process. We have already reported on the analysis of the hepatocyte transcriptome^12^. Here, we focused our detailed analysis on the LSECs and KCs since previous transcriptional studies on these NPCs in an *in vitro* context have been limited. We related our results, which are based on transcriptional data, to published *in vivo* or *in vitro* experiments.

Just like hepatocytes, LSECs require communication with other hepatic cells for normal physiological phenotype and function^9,94^. Phenotypic characteristics of LSECs include the presence of open fenestrae, which are associated with the endocytic capabilities of LSECs^2^. Maintaining this phenotype *in vitro* is challenging since LSECs rapidly dedifferentiate within days^36,100^. We have demonstrated earlier that fenestrae found in healthy LSECs disappear in the presence of activated KCs^101^. To the best of our knowledge, the current work is the first to provide clear transcriptional evidence that suggests that LSECs maintain a differentiated phenotype when cultured in a tri-cellular, organotypic hepatic environment.

Nitric oxide (NO) is one of the key factors that are involved in the maintenance of LSEC phenotype^36^. Endothelial NO synthase (eNOS; Nos2) is a primary contributor to the production of NO in LESCs^2^. LSECs in the 3DHLK model show increased levels of eNOS gene expression compared to those in the 3DHL model. There are several ways in which eNOS may be activated in LSECs; our data contains evidence to support two such mechanisms. First, Nicotinic and Muscarinic receptor activation leads to active eNOS in endothelial cells^39^ via increased cytosolic calcium ion levels as a result of opened ion channels^37,38^. We observed up-regulation of ion channel, neurotransmitter receptor, and GPCR activity, all functions involving the Nicotinic and Muscarinic receptors. Second, vascular endothelial growth factor A (Vegfa) promotes the maintenance of fenestrae by inducing eNOS activity and subsequent production of NO^2,36^. In culture and *in vivo*, Vegfa has been shown to be sufficient in maintaining LSECs fenestration^102^. Since KCs are known to express Vegfa^103^, it is possible that LSECs may be exposed to additional Vegfa originating from the KCs in the 3DHLK model. Further mechanistic experiments will need to be performed to identify the direct cause of this amplified eNOS expression. Nevertheless, our results are consistent with LSECs in the 3DHLK model producing increased levels of NO, thereby exhibiting a fenestrated phenotype.

*In vivo*, a decrease in the numbers and diameter of fenestrae on LSECs is often associated with fibrosis^2^. In addition, increased focal adhesions have been correlated with a loss of fenestrae *in vitro*^101^. LSECs in the 3DHLK model exhibited decreased FAK signaling. Three participants in FAK signaling, Rock2^32^, RhoA^33^ and Rac1^34^, have all been associated with negatively regulating LSEC fenestration. We observe decreased expression of Rock2 and Rac1 in LSECs cultured in the presence of KCs, i.e., in the 3DHLK model. Rock2 has been found to negatively regulate eNOS activity in cirrhosis^104^. The decreased expression of Rock2 may provide an additional explanation for the observed increased expression of eNOS in LSECs cultured in the presence of KCs., thus providing additional evidence for LSEC fenestration in the 3DHLK model. Taken together, these results support the conclusion that the 3DHLK liver model provides a non-fibrotic environment for LSECs.

PPARα activity has extensively been studied in hepatocytes^56,105–107^ with respect to its role in lipid metabolism^56,106^, insulin sensitivity^56^, and its target genes^107^. Altered PPARα signaling has also been linked to fatty liver disease^108^, obesity^109^, and diabetes. However, considerably less information is present for its gene expression or protein activity in LSECs. Several non-hepatic ECs have exhibited Ppara expression^110,111^, but a recent study in female murine liver ECs failed to detect significant Ppara mRNA^112^. In contrast, we observed that LSECs in the 3DHLK model express Ppara, and at higher levels than LSECs in the 3DHL model and hepatocytes from both 3D cultures. In the liver, PPARα is known to transcriptionally control gene sets related to fatty acid metabolism^113^, bile acid biosynthesis^58–60^, amino acid degradation^63^, and cholesterol metabolism^57^, phenomena that are mirrored in our LSEC-specific data. Our novel results indicate that PPARα may have a pivotal role in LSECs as a regulator of many biological processes that are critical to proper hepatic function.

PPARα transcriptionally up-regulates Cyp7a1, Cyp8b1 and Cyp27a1^58–60^, which are responsible for maintaining the pool of bile acids (BAs)^58,76^. Studies have shown that the bile acid chenodeoxycholic acid (CDCA) induces FXR activity in humans and mice^76,77^. In human HepG2 cells, activation of FXR by CDCA has been associated with increased expression of PPARa^78^. We found transcriptional support for this positive feedback mechanism between PPARα and FXR in LSECs in the 3DHLK model (Fig. 3b). Our analysis confirms the up-regulation of Cyp7a1, Cyp8b1 and Cyp27a1 in LSECs from the 3DHLK model, which may be the result of increased PPARα activity as suggested by the functional link between PPAR Signaling and Terpenoid Backbone Biosynthesis in the BPN (Fig. 2a,c). Conversely, FXR is known to activate Nuclear Receptor Subfamily 0, Group B, Member 2 (SHP; Nr0b2), another nuclear receptor, which in turn inhibits Cyp7a1^58^ (Fig. 3b). FXR and SHP showed increased expression in LSECs in the 3DHLK model. Taken together, these transcriptional results indicate that LSECs may balance the positive and negative feedback among PPARα, FXR, and BA biosynthesis, mechanisms which were previously observed in HepG2 cells.

Bile acid biosynthesis is a well known function of the liver^58^. We have previously shown that 3DHL models synthesize and metabolize bile acids^74^. Here, we find transcriptional support for altered bile acid metabolism in LSECs cultured in the organotypic liver models. Perturbed bile acid metabolism in LSECs may stem from the uptake of BAs originating at hepatocytes in the 3DHLK model. Solute Carrier Family 10 Member 1 (known as Slc10a1 or NTCP), a membrane transporter responsible for the influx of extracellular BAs, has been shown to be functionally present in hepatocytes^114^. In our data, this transporter shows higher expression levels in LSECs compared to hepatocytes from the same liver model (3DHLK or 3DHL). Moreover, Slc10a1 also shows a marked increase in expression in LSECs in the presence of KCs, i.e., in 3DHLK models. If LSECs in the 3DHLK model uptake BAs, then exogenous CDCA may activate the FXR receptor and cause transcriptional up-regulation of bile acid biosynthesis enzymes through the feedback mechanisms discussed earlier (Fig. 3b). Further mechanistic experiments will need to be performed to understand the precise role of Slc10a1 and exogenous BAs in the feedback loop between PPARα and FXR.

KCs, like other macrophages, display a continuum of phenotypes from proinflammatory (M1) to anti-inflammatory (M2)^92,93,115^. Under proinflammatory conditions, activated KCs release cytokines (e.g., Tnf-α, IL1, and IL6), chemokines, and reactive oxygen species^93^. In contrast, M2 KCs express IL10, IL1 receptor agonist, and IL1 decoy receptor^99^. Activated KCs have been implicated in liver fibrosis in non-alcoholic steatohepatitis mouse models^116^, whereas anti-inflammatory KCs promote tissue repair and suppress inflammation^117^. KCs in 3D cultures show decreased expression of proinflammatory cytokines, indicating a more M2-like phenotype and suggesting that the 3DHLK model provides a healthy (non-fibrotic) environment for culturing hepatic cells.

It is known that, in addition to proper *in vivo* architecture, intercellular communications are essential for the maintenance of phenotype and function of hepatic cells^9,94^. Despite the importance of these heterotypic signals originating in NPCs, transcriptional studies of the liver have mainly focused on hepatocytes^5–7,9,14–16^. Here, we provide the first genome-wide transcriptional study of hepatic NPCs in an organotypic liver model. Through an extensive functional enrichment and network analysis of these transcriptional data, we have shown that LSECs in the 3DHLK model maintain their differentiation and that KCs present with an anti-inflammatory phenotype. These results demonstrate the 3DHLK model represents a more *in vivo*-like environment for LSECs and KCs than 3DHL models. Our computational analysis has uncovered previously unknown events in LSECs, including the expression of PPARα and genes that participate in the processes it regulates. We also hypothesize that the feedback mechanisms that connect PPARα, FXR, and BA biosynthesis, previously identified in HepG2 cells, may also occur in LSECs. We note that our analyses are based on transcriptional data. Hence, further mechanistic experiments are required to confirm our results. Nevertheless, our work calls attention to the potential role LSECs may play in the pathogenesis of diseases such as obesity and diabetes. These discoveries emphasize the need for further study of the functions and activities of hepatic non-parenchymal cells both *in vitro* and *in vivo*.

## Materials and Methods

### Materials

Dulbecco’s modified Eagle’s medium (DMEM), phosphate-buffered saline (PBS), Hank’s buffered salt solution, Earle’s balanced salt solution, penicillin, streptomycin, human plasma fibronectin, and trypsin-ethylenediaminetetraacetic acid were purchased from Invitrogen Life Technologies, Carlsbad, CA. Type IV collagenase, HEPES (4-[2-hydroxyethyl] piperazine-1-ethanesulfonic acid), glucagon, calcium chloride, hydrocortisone, sodium dodecyl sulfate, hydrogen peroxide, glutaraldehyde, dicumarol, 3-methylcholanthrene, calf thymus DNA, chitosan, and hyaluronic acid (HA) was purchased from Sigma-Aldrich, St. Louis, MO. All other chemicals were purchased from Fisher Scientific, Waltham, MA, unless otherwise noted.

### Assembly of detachable polymeric Space of Disse

Detachable polyelectrolyte multilayers (PEMs) were assembled using chitosan (cationic) and HA (anionic) as previously described^12^. Briefly, chitosan was dissolved in 1% v/v acetic acid and HA in 18 MΩ cm water (Hydro, Durham, NC). Their concentrations were 5 mM. The pH of the polyelectrolyte (PE) solutions was maintained at 4.0 and 5.0 for chitosan and HA, respectively. PEMs were assembled on hydrophobic poly-tetrafluoroethylene (PTFE; McMaster-Carr, Elmhurst, IL) substrates using a robotic deposition system (NanoStrata, Tallahassee, FL). Water contact angle values (KSV Instruments, Helsinki, Finland) on clean PTFE substrates ranged from 111.9 ± 4.2 (n = 15). A detailed study was performed to find the optimal number of bilayers (BLs), deposition times, optical properties and the Young’s modulus for PEMs^12^. 12.5 BLs with a deposition time of 40 min have previously been reported to provide stable detachable PEMs with properties similar to the Space of Disse^12^.

### Isolation and culture of hepatocytes and LSECs and KCs

Primary hepatocytes were isolated from female Lewis rats (Harlan Laboratories, Indianapolis, IN; weighing 175–199 g) utilizing a two-step *in situ* collagenase perfusion method^5,6,9,10,118^. Hepatocyte yields ranged from 150–200 × 10^6^ cells and their viability was determined to be 90%–95% based on trypan blue exclusion. LSECs, from the same isolation, were obtained using differential adhesion and were cultured on fibronectin coated flasks^9^. Cryopreserved primary rat KCs (Invitrogen Life Technologies) were maintained in DMEM supplemented with 10% (v/v) heat-inactivated fetal bovine serum, 100 U/mL penicillin, 100 μg/mL streptomycin, 10 μg/mL insulin, and 100 μM β-mercaptoethanol. Animal care and surgical procedures including the procedure for liver excision from rats were conducted in accordance with the guidelines and regulations set up by the Virginia Tech’s Institutional Animal Care and Use Committee. All cell culture work was approved by Virginia Tech’s Institutional Biosafety Committee.

### Assembly of 3D multicellular hepatic cultures

Hepatocytes were initially cultured as monolayers up to 72 h in 12-well tissue culture polystyrene plates (BD Biosciences, San Jose, CA) coated with rat-tail type 1 collagen (BD Biosciences)^9,10^. For the 3D liver models, UV-sterilized detachable PEMs (12.5 BL) were placed above the layer of hepatocytes. The PEMs were hydrated in the presence of hepatocyte culture medium for 1 h. Thereafter, 25,000 LSECs were seeded on the PEM. In cultures containing KCs, initially 50,000 cells were seeded on the PEM to obtain an initial ratio of 10:1 hepatocytes:KCs to emulate healthy livers^119^. All multicellular cultures were maintained in hepatocyte culture medium.

### Gene chip hybridization and microarray data quality control

Hepatocytes, LSECs and KCs were separated as previously reported^12^. Briefly, 3D liver models were exposed sequentially to Dynabeads^R^ (CELLection^TM^ Kit; Invitrogen Life Technologies) coated with SE-1 and CD163 antibodies^120^. SE-1 and CD163 are LSEC- and KC-specific antibodies, respectively. LSEC and KC fractions were collected using a magnet (DynaMag^TM^; Invitrogen Life Technologies). Total RNA was extracted from each cell type from HM, CS, and 3DHL and 3DHLK on day 12, and KC monolayers on day 3 using an RNeasy mini kit (Qiagen, Germantown, MD). The samples were checked for RNA degradation based on the RNA Integrity Number^121^. Each cell type and model combination was performed in triplicates, except KC samples from 3DHLK on day 12 which had only two samples that passed the RNA quality metric. Samples were labeled according to the Affymetrix Standard Target labeling process, and hybridized to the GeneChip Rat Genome 230 2.0 (Affymetrix, Santa Clara, CA). Complementary RNA synthesis, hybridization, and GeneChip scanning were performed at the Virginia Bioinformatics Institute Core Laboratory.

### Gene expression analysis

We processed the Affymetrix microarrays using the Robust MultiChip Average (RMA) method^122^ in R. RMA performs background correction, quantile normalization and probe summarization. Each of the microarrays met the quality standards recommended by Simpleaffy^123^. As a second quality control, we performed hierarchical clustering on the normalized data to show consistency between replicates (Fig. 1). We performed hierarchical clustering on our gene expression data using complete linkage and Euclidian distance^30^. We used a data-driven approach to analyze the gene expression data further, which we describe in the next two subsections.

### Functional enrichment

To compare the functional differences between LSECs in the 3DHLK and 3DHL models at day 12, we performed functional enrichment on the normalized gene expression data using the Gene Set Enrichment Analysis (GSEA) package^31^. GSEA summarizes probe-level data into gene-level data, computes differential expression of each gene using the signal-to-noise ratio (SNR), and calculates the enrichment of each functional gene set. To obtain the null distribution of *p*-values, GSEA permutes gene sets 5000 times. GSEA estimates the false discovery rate (*q*-value) using the method of Benjamini and Hochberg^124^. We considered gene sets with an FDR *q*-value ≤ 0.01 as significant. For each enriched gene set, GSEA also computed the leading edge, defined as the subset of genes annotated to the gene set that contributed to its enrichment. We note that for the KCs in the 3DHLK model, we simulated a third sample to be the mean of the other two samples.

We used gene sets from the Molecular Signature Database (MSigDB) v4.0^31^ and Netpath^125^, a curated resource for signal transduction pathways. To enable the detection of perturbed biological processes and pathways, we included C2:CP and C5 gene sets in the analysis. The C2:CP collection contained pathways taken from databases such as Biocarta (a resource that is no longer available independently), KEGG^126^, and Reactome^127^, publications in PubMed, and knowledge of domain experts. C5 gene sets corresponded to annotations to Gene Ontology (GO) terms^128^. NetPath provides three gene sets for each pathway: (1) the genes directly involved in the pathway, (2) the genes transcriptionally up-regulated as a result of activating the pathway, and (3) the genes transcriptionally down-regulated by the pathway. Since very small or very large gene sets may be hard to interpret, we filtered out gene sets with less than 10 or more than 500 member genes, leaving 2501 gene sets for functional enrichment analysis.

### Biological process networks

We sought to better understand the relationship among the significantly enriched gene sets reported by GSEA by integrating the gene expression data with protein interaction networks. Our goal was to compute a network where nodes were gene sets and where links connected pairs of gene sets, with each link being supported by multiple interactions between genes annotated to each set. To this end, we used a previously-published algorithm: Markov chain Monte Carlo Biological Process Networks (MCMC-BPN)^45^ to link the significantly enriched gene sets reported by GSEA. MCMC-BPN computes a network whose nodes are gene sets and whose links connect gene sets (e.g., Fig. 2a). The algorithm uses a Markov Chain Monte Carlo process to select as few links between genes as possible that explain as many interactions as possible between perturbed genes annotated to different gene sets. In our earlier work, we have demonstrated that MCMC-BPN produces sparser and less redundant networks than competitive methods without sacrificing coverage of the protein interaction network^45.^

MCMC-BPN takes as input a protein  interaction network, a collection of gene sets, and a set of perturbed genes. We created these inputs as follows:

- We downloaded a rat functional interaction network from STRING^129^ on January 27, 2015. Each interaction (u,v) in this network has a score computed by STRING. To obtain a reliable network, we removed every interaction with a score less than 500. Further, we retained only those interactions (u,v) such that both u and v were present on the DNA microarray. The final network contained 7,994 nodes and 70,052 undirected interactions.
- We used the gene sets deemed significant by GSEA (*q*-value ≤ 0.01).
- We defined a gene to be *perturbed* if that gene participated in the leading edge of at least one significant gene set in the previous step. The final input contained all such genes.

We ran MCMC-BPN with a burn-in period of 10,000,000 steps followed by 100,000,000 MCMC steps.

### Literature analysis

Until this stage, our analysis was driven completely by gene expression data, functional annotation of genes, and gene interaction networks. We examined the BPNs computed, manually selected gene sets or groups of gene sets from these networks, and used the published literature on these gene sets in the liver for discussion in the paper.

### Data availability

The microarray datasets generated during the current study are available in the Gene Expression Omnibus repository at http://www.ncbi.nlm.nih.gov/geo/query/acc.cgi?acc=GSE74424.

## Electronic supplementary material

Table S1
